# Retrospective Cohort Study of Pregnancy Maternal Outcomes of Women With COVID‐19 in King Salman Armed Forces Hospital, Tabuk, Kingdom of Saudi Arabia

**DOI:** 10.1155/ogi/3287655

**Published:** 2025-12-22

**Authors:** Abdulrahman Wasel Koja, Rofayda Mansour Ahmed Mohamad, Mubarak Saleh Almarjan, Mayar Hamed Albakri

**Affiliations:** ^1^ Preventive Medicine Department, Ministry of Health, Tabuk, Saudi Arabia, moh.gov.sa; ^2^ Preventive Medicine Department, King Salman Armed Forces Hospital, Tabuk, Saudi Arabia, nwafh.med.sa; ^3^ Obstetrics and Gynecology Department, King Abdulaziz Hospital, Jeddah, Saudi Arabia, ngha.med.sa

**Keywords:** comorbidity, COVID-19, newborns, outcomes, pregnant women, SARS-CoV-2, Saudi Arabia, severity

## Abstract

**Aim:**

This study aimed to evaluate the adverse maternal and neonatal outcomes among pregnant women with COVID‐19 at King Salman Armed Forces Hospital in Tabuk and examine the relationship between the severity of COVID‐19 infection and these outcomes.

**Methods:**

This retrospective cohort study enrolled COVID‐19–positive pregnant women. Demographic and obstetric information, clinical characteristics (including symptoms and preexisting comorbid conditions), and maternal and neonatal outcomes were collected from medical records, reviewed, and analyzed.

**Results:**

The study included 138 COVID‐19–positive women, with the majority of cases diagnosed in the third trimester (85.5%). Assessment of the severity of COVID‐19 infection showed that a large proportion were asymptomatic (39.1%) or had mild disease (39.9%), while 14.5% had moderate disease and 6.5% experienced severe illness. Adverse maternal and neonatal outcomes included preeclampsia (5.1%), gestational diabetes (4.3%), placental abruption, premature rupture of membranes (2.9% each), maternal intensive care unit (ICU) admission (1.4%), preterm births (12.3%), low birth weight (15.2%), and neonatal ICU admission (10.1%). Additionally, the incidences of preeclampsia and maternal ICU admission were significantly higher in women with severe COVID‐19 compared to those with milder or asymptomatic cases (*p* = 0.004 each). Neonatal ICU admission showed the highest incidence (33.3%) among severe cases (*p* = 0.031). Asthma was significantly linked to a higher risk of neonatal ICU admission (23.1% vs. 7.1%). Diabetes mellitus was associated with an increased rate of maternal ICU admission (13.3% vs. 0%). Hypertension showed significant associations with elevated rates of preeclampsia (35.7% vs. 1.6%), maternal ICU admission (14.3% vs. 0%), and neonatal ICU admission (35.7% vs. 7.3%).

**Conclusion:**

COVID‐19 infection during pregnancy was associated with several adverse maternal and neonatal outcomes, including preeclampsia, gestational diabetes, preterm birth, low birth weight, and increased ICU admissions. Severe maternal disease and existing comorbidities further elevated the risks of complications for both mother and newborn.

## 1. Introduction

The impact of the novel coronavirus disease 2019 (COVID‐19) on global health systems has been profound since its emergence in late 2019. Among the populations at increased risk, pregnant women have received particular attention due to physiological changes during pregnancy, which increase susceptibility to infections [1, 2].

Growing evidence suggests that COVID‐19 infection during pregnancy may be associated with a range of maternal, fetal, and neonatal complications [3]. Several case series have demonstrated that COVID‐19 infection during pregnancy is linked to severe maternal infection, increased risk of maternal morbidity and mortality, and spontaneous abortion. There have also been reports of preterm labor, intrauterine fetal death, low birth weight, and increased neonatal intensive care unit (ICU) admissions [4–8].

Exploring the association of COVID‐19 severity with adverse maternal outcomes is essential for improving clinical management, guiding resource allocation, and establishing evidence‐based protocols for care during the pandemic.

Saudi Arabia has experienced several COVID‐19 waves, affecting vulnerable groups, including pregnant women. A few studies have contributed valuable insights into how the coronavirus impacts pregnant women, revealing a range of clinical manifestations, pregnancy complications, and neonatal outcomes [9–11].

This study addresses the lack of region‐specific data on COVID‐19 in pregnancy in Saudi Arabia, particularly the northwestern region. It provides hospital‐based findings from King Salman Armed Forces Hospital in Tabuk to support national health data and guide targeted maternal and child health policies. The study aimed to assess adverse maternal and neonatal outcomes in COVID‐19–positive pregnant women and explore how disease severity closely relates to these outcomes.

## 2. Materials and Methods

### 2.1. Study Design, Settings, and Date

This retrospective cohort study was conducted at King Salman Armed Forces Hospital, Tabuk, Saudi Arabia, between December 2024 and February 2025. King Salman Armed Forces Hospital in Tabuk is a leading JCI‐accredited medical institution with over 1000 beds. It offers comprehensive healthcare services, serves as a teaching hospital, and is accredited for multiple residency programs and fellowships.

The study utilized data retrieved from the electronic medical records of pregnant women with laboratory‐confirmed severe acute respiratory syndrome coronavirus 2 (SARS‐CoV‐2) infection at the hospital. The study aimed to evaluate adverse maternal and neonatal outcomes associated with COVID‐19 infection and examine their relationships with disease severity and preexisting comorbidities. Measurable outcomes included preeclampsia, gestational diabetes, preterm birth, premature rupture of membranes, mode of delivery, maternal ICU admission, neonatal birth weight, Apgar scores, and neonatal ICU admission.

### 2.2. Inclusion Criteria

The study included pregnant women of any gestational age who received care at King Salman Armed Forces Hospital between January 1, 2021, and December 31, 2022, with confirmed SARS‐CoV‐2 infection by real‐time reverse transcriptase polymerase chain reaction (RT‐PCR) test.

### 2.3. Exclusion Criteria

Patients with incomplete medical records or who were transferred to other facilities before delivery, and women with multiple pregnancies were excluded.

### 2.4. Sample Size and Sampling Technique

Based on the eligibility criteria, we estimated the sample size to be approximately 195–210 COVID‐19–positive pregnant women. This estimation considered potential exclusions due to incomplete medical records or transfers to other facilities. A comprehensive sampling approach was employed, including all eligible cases within the specified study period.

### 2.5. Outcomes

The primary outcome in this study was the maternal outcome, defined as any adverse maternal event occurring during pregnancy, delivery, or within 42 days postpartum in women with confirmed SARS‐CoV‐2 infection. This included pregnancy complications such as preeclampsia, gestational diabetes, preterm labor, premature rupture of membranes, postpartum hemorrhage, severe maternal morbidity requiring ICU admission, mode of delivery (vaginal vs. cesarean section), and preterm birth (delivery before 37 weeks of gestation).

The secondary outcome included neonatal birth weight, Apgar score at 1 and 5 min, and the need for neonatal ICU admission.

### 2.6. Treatment

COVID‐19 patients received treatment following World Health Organization guidelines. All additional management and procedures were carried out based on obstetric indications and the hospital’s standard protocols.

### 2.7. Data Collection

Data were extracted from the electronic medical records using a standardized data collection form developed by the research team. The form was designed to capture demographic information such as the maternal age, occupation, education level, and marital status; obstetric history including gravidity, parity, previous cesarean section; medical history for any preexisting chronic comorbidities such as diabetes mellitus, hypertension, asthma, obesity, and thyroid disorders; COVID‐19 symptoms and severity (based on WHO criteria); received treatment; and outcomes including maternal or pregnancy complications, maternal ICU admission, mode and time of delivery, neonatal Apgar scores at 1 and 5 min, birth weight, and the need for neonatal ICU admission. All cases meeting the inclusion criteria between January 2021 and December 2022 were reviewed. Data accuracy was ensured through double entry by two independent investigators, with discrepancies resolved through discussion.

### 2.8. Statistical Analysis

All data were analyzed by the statistical package SPSS (Statistical Package for the Social Sciences) Version 27 (IBM Corp., Armonk, NY, USA). Descriptive statistics included summarization of categorical data as numbers and percentages, as well as numerical data, which were first tested for normality by the Shapiro–Wilk test, Q–Q plot, and histograms. The normally distributed numerical data were expressed as mean ± standard deviation, whereas skewed data were displayed as median and interquartile range. The inferential statistics involved the associations between the maternal and neonatal outcomes as dependent variables and the independent variables, including preexisting morbidities and severity of COVID‐19 infection. These were performed using the chi‐square, Fisher’s exact, or Fisher–Freeman–Halton exact tests as appropriate. When the Fisher–Freeman–Halton exact result was significant, it was followed by the post hoc *Z*‐test to show the categories with significant differences between the studied groups. The maternal age was also considered an independent determinant of the study outcomes. Comparisons of the distributions of maternal age between different groups were performed by the Mann–Whitney *U* test. A *p* value < 0.05 was considered statistically significant.

### 2.9. Ethical Considerations

This study was conducted in accordance with the Declaration of Helsinki and Good Clinical Practice guidelines. Ethical approval was obtained from the Research Ethics Committee of King Salman Armed Forces Hospital, Tabuk, Saudi Arabia. Given the retrospective nature of the study, the requirement for informed consent was waived. All patient data were deidentified prior to analysis, and access to medical records was restricted to the research team. Data were stored in password‐protected files to ensure confidentiality.

## 3. Results and Discussion

### 3.1. Results

The study included 138 COVID‐19–positive women. Table 1 presents baseline characteristics of the studied COVID‐19–positive women (*N* = 138). Most COVID‐19 infections occurred in the third trimester (85.5%). The mean maternal age was 31.1 ± 7.4 years. Most participants (42.0%) had attained university education and were housewives (43.5%). Previous cesarean sections were reported by 42.8%. Preexisting comorbidities included obesity (22.5%), diabetes mellitus (19.9%), asthma (18.8%), hypertension (10.1%), and thyroid disorders (5.1%).

**Table 1:** Sociodemographic, obstetric, and preexisting comorbid characteristics of the studied COVID‐19–positive pregnant women.

	**COVID-19**–**positive pregnant women (*N* = 138)**

Maternal age, years, median (IQR)	30.0 (25.0–36.0)
First trimester (1–12 weeks), *n* (%)	4 (2.9%)
Second trimester (13–26 weeks), *n* (%)	16 (11.6%)
Third trimester (27 weeks to birth), *n* (%)	118 (85.5%)
Education level, *n* (%)	
Primary	4 (2.9%)
Secondary	49 (35.5%)
University	58 (42.0%)
Postgraduate	27 (19.6%)
Occupation, *n* (%)	
Housewife	60 (43.5%)
Employed	41 (29.7%)
Unemployed	19 (13.8%)
Student	18 (13.0%)
Marital status, *n* (%)	
Married	133 (96.4%)
Divorced	5 (3.6%)
Widowed	0 (0.0%)
Gravidity (median, IQR)	3.0 (2.0–5.0)
Parity (median, IQR)	2.0 (1.0–3.0)
Previous cesarean section, *n* (%)	
No	79 (57.2%)
One	32 (23.2%)
Two	18 (13.0%)
More than two	9 (6.5%)
Preexisting comorbidities, *n* (%)	
Asthma	26 (18.8%)
Diabetes mellitus	15 (10.9%)
Hypertension	14 (10.1%)
Obesity	31 (22.5%)
Thyroid disorders	7 (5.1%)

*Note: N*, number.

Abbreviation: IQR, interquartile range.

**Figure 1:**
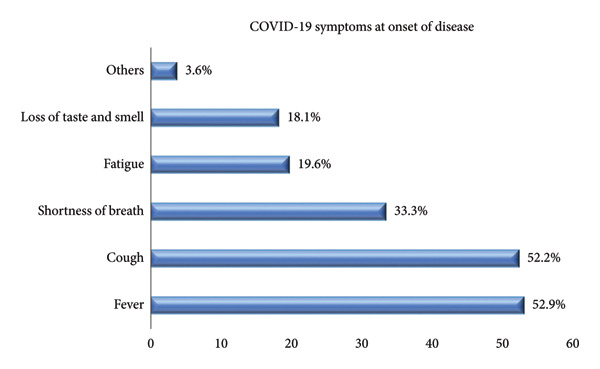
Distribution of symptoms at the onset of the COVID‐19.

**Figure 2:**
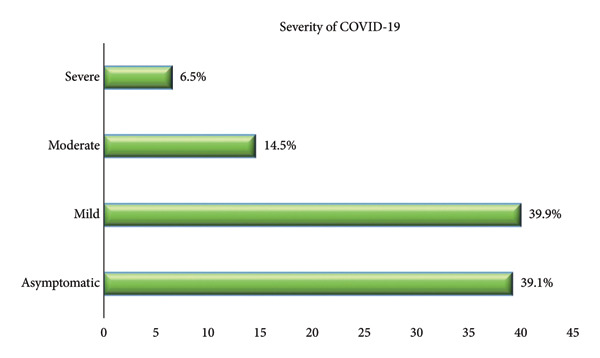
Distribution of severity of the COVID‐19.

The most commonly reported symptoms at COVID‐19 onset were fever (52.9%) and cough (52.2%), followed by shortness of breath (33.3%) and fatigue (19.6%). Additionally, loss of taste and smell was experienced by 18.1% of the women. Regarding the severity of COVID‐19, a large proportion of the cases were either asymptomatic (39.1%) or presented with mild symptoms (39.9%), whereas 14.5% had moderate disease and 6.5% experienced severe illness (Figures 1 and 2).

The identified adverse maternal and neonatal outcomes among the COVID‐19–positive women included preeclampsia (5.1%), gestational diabetes (4.3%), placental abruption, premature rupture of membranes (2.9% each), maternal ICU admission (1.4%), preterm births (12.3%), low birth weight (15.2%), and neonatal ICU admission (10.1%). Moreover, the mean Apgar scores at 1 and 5 min were 8.1 ± 1.0 and 8.5 ± 1.0, respectively, and the mean length of hospital stay was 4.1 ± 2.5 days (Table 2).

**Table 2:** The incidence of maternal and neonatal outcomes among the studied COVID‐19–positive pregnant women.

		**COVID-19**–**positive pregnant women (*N* = 138)**

Pregnancy complications, *n* (%)	Preeclampsia	7 (5.1%)
	Eclampsia	0 (0.0%)
	Gestational diabetes	6 (4.3%)
	Placental abruption	4 (2.9%)
	PROM	4 (2.9%)
	None	117 (84.8%)

Maternal ICU admission, *n* (%)	No	136 (98.6%)
	Yes	2 (1.4%)

Mode of delivery, *n* (%)	Cesarean section	73 (52.9%)
	Vaginal	65 (47.1%)

Preterm birth (before 37 weeks of gestation), *n* (%)	No	121 (87.7%)
	Yes	17 (12.3%)

Low birth weight (less than 2500 g), *n* (%)	No	117 (84.8%)
	Yes	21 (15.2%)

Neonatal ICU admission, *n* (%)	No	124 (89.9%)
	Yes	14 (10.1%)

Apgar score at 1 min, median (IQR)		8.0 (7.0–8.0)

Apgar score at 5 min, median (IQR)		9.0 (8.0–9.0)

Duration of hospital stay (days), median (IQR)		3.0 (2.0–4.0)

*Note: N*, number.

Abbreviations: ICU, intensive care unit; IQR, interquartile range; PROM, premature rupture of membranes.

Table 3 shows the associations between the severity of COVID‐19 and adverse maternal and neonatal outcomes in pregnant women (*N* = 138). The incidence of preeclampsia and maternal ICU admission was significantly higher in women with severe COVID‐19 compared to those with milder or asymptomatic cases (*p* = 0.004 for both). Preeclampsia occurred in 33.3% of severe cases versus only 1.9% of asymptomatic cases and 1.8% of mild cases. Similarly, maternal ICU admission was reported in 22.2% of severe cases, while no ICU admissions occurred among asymptomatic, mild, or moderate cases. Neonatal ICU admission also showed the highest incidence (33.3%) among severe cases (*p* = 0.031).

**Table 3:** Associations between the severity of COVID‐19 and the frequency of adverse maternal and neonatal outcomes in pregnant women (*N* = 138).

		**COVID-19 severity**				**p** **value**
		**Asymptomatic**	**Mild**	**Moderate**	**Severe**	

Pregnancy complications, *n* (%)	Preeclampsia	1 (1.9%)	1 (1.8%)	2 (10.0%^a^)	3 (33.3%^a^)	0.004^∗^
	Gestational diabetes	2 (3.7%)	2 (3.6%)	2 (10.0%)	0 (0.0%)	
	Placental abruption	0 (0.0%)	1 (1.8%)	2 (10.0%)	1 (11.1%)	
	PROM	2 (3.7%)	1 (1.8%)	1 (5.0%)	0 (0.0%)	
	None	49 (90.7%^a^)	50 (90.9%^a^)	13 (65.0%)	5 (55.6%)	

Mode of delivery, *n* (%)	Cesarean section	23 (42.6%)	29 (52.7%)	14 (70.0%)	7 (77.8%)	0.081
	Vaginal	31 (57.4%)	26 (47.3%)	6 (30.0%)	2 (22.2%)	

Preterm birth (before 37 weeks of gestation), *n* (%)	No	46 (85.2%)	49 (89.1%)	19 (95.0%)	7 (77.8%)	0.474
	Yes	8 (14.8%)	6 (10.9%)	1 (5.0%)	2 (22.2%)	

Low birth weight (less than 2500 g), *n* (%)	No	45 (83.3%)	48 (87.3%)	17 (85.0%)	7 (77.8%)	0.797
	Yes	9 (16.7%)	7 (12.7%)	3 (15.0%)	2 (22.2%)	

Maternal ICU admission, *n* (%)	No	54 (100.0%)	55 (100.0%)	20 (100.0%)	7 (77.8%)	0.004^∗^
	Yes	0 (0.0%)	0 (0.0%)	0 (0.0%)	2 (22.2%^a^)	

Neonatal ICU admission, *n* (%)	No	50 (92.6%)	52 (94.5%)	16 (80.0%)	6 (66.7%)	0.031^∗^
	Yes	4 (7.4%)	3 (5.5%)	4 (20.0%)	3 (33.3%^a^)	

^∗^Significant at *p* < 0.05, superscript letter.

^a^Categories that showed significant differences between the studied groups.

Supporting Table 1 demonstrates no significant differences in maternal age among different maternal and neonatal outcomes (*p* values > 0.05), except for preeclampsia (*p* = 0.004) and mode of delivery (*p* = 0.008), with older women more likely to develop preeclampsia or undergo cesarean delivery.

Supporting Table 2 shows that asthma was significantly associated with an increased risk of neonatal ICU admission (23.1% vs. 7.1%). Diabetes mellitus was also significantly associated with a higher rate of maternal ICU admission (13.3% vs. 0%). Hypertension comorbidity was significantly associated with an increased incidence of preeclampsia (35.7% vs. 1.6% in those without hypertension), maternal ICU admission (14.3% vs. 0%), and neonatal ICU admission (35.7% vs. 7.3%). Obesity did not show significant associations with most adverse outcomes, though there was a significant trend toward higher rates of placental abruption in obese women (9.7% vs. 0.9%).

### 3.2. Discussion

This study enrolled 138 COVID‐19–positive pregnant women to determine the patterns of complications associated with COVID‐19 infection.

Most COVID‐19–positive patients were diagnosed during the third trimester. This finding aligns with previous studies [12–15] and may be due to a stronger inflammatory response in the first and second trimesters that may lower infection risk [16]. The most frequent early symptoms were fever, cough, fatigue, shortness of breath, and loss of taste or smell, which agrees with previous studies [9, 12, 17, 18].

The detected adverse maternal and neonatal outcomes included preeclampsia, gestational diabetes, placental abruption and PROM, maternal ICU admission, preterm births, low birth weight, and neonatal ICU admission. These findings coincide with the commonly reported association between the COVID‐19 infection and worse maternal and neonatal outcomes [10, 19–22]. However, a study in Iran revealed no significant relationship between COVID‐19 infection and various maternal or neonatal complications [23].

Recent international meta‐analyses have confirmed that COVID‐19 infection increases the risk of preeclampsia, preterm birth, and neonatal ICU admission, highlighting the global consistency of these findings [24–28]. Regionally, studies from Jazan and Riyadh, Saudi Arabia, reported similar adverse outcomes [9, 10].

The lower incidence of COVID‐19–related complications in this study, compared to earlier reports, might be attributed to the low frequency of severe COVID‐19 infection. In addition, close monitoring and more frequent medical care of the COVID‐19–infected women could potentially lead to fewer complications. It is also important to note that the virulence of SARS‐CoV‐2 variants has evolved, with later strains generally associated with milder disease and fewer complications [29].

Regarding the severity of infection, most COVID‐19 patients were either asymptomatic (39.1%) or exhibited mild symptoms (39.9%), whereas 14.5% developed moderate illness and 6.5% suffered from severe disease. Similarly, Dileep et al. [30] found that the most COVID‐19–infected pregnant women had mild symptoms (73.5%) and only 26.5% had moderate to severe manifestations. Also, a previous study reported 86.0% mild cases and around 14.0% between severe and critical cases [31]. Some earlier studies have suggested that COVID‐19 infection may not be more severe in pregnant women compared to the general population [32–34].

Severe COVID‐19 in pregnancy was significantly associated with a higher risk of serious maternal complications and adverse neonatal outcomes, particularly preeclampsia, maternal ICU admission, and neonatal ICU admission. Similarly, Dileep et al. [30] reported that pregnant women with moderate to severe COVID‐19 were at much higher risk of preterm delivery, lower birth weight, neonatal infection, and neonatal ICU admission. Other studies indicated an increased risk of adverse obstetric and neonatal outcomes such as admission to the ICU and the need for cesarean delivery among pregnant women with severe COVID‐19 [20, 35, 36]. Furthermore, Sami et al. [15] in the United Arab Emirates concluded significant differences regarding ICU admission, maternal mortality, emergency cesarean section, and low birth weight in relation to severity.

In this study, older women were associated with a higher incidence of preeclampsia and a higher frequency of cesarean delivery. Previous studies emphasized that older maternal age is a well‐known risk factor for preeclampsia possibly due to age‐related changes in vascular function, endothelial health, and metabolic profile [37], and cesarean section [38].

Maternal comorbidities in this study were linked to adverse outcomes for both mothers and newborns. Asthma increased neonatal ICU admissions, diabetes raised maternal ICU admissions, and hypertension was associated with preeclampsia and both maternal and neonatal ICU admissions. While obesity was not strongly linked to most adverse outcomes, it showed a higher tendency for placental abruption. Previous studies [3, 20, 39, 40] reported similar findings. These results emphasize the importance of comprehensive prenatal screening and individualized care plans for pregnant women with comorbidities.

From a public health perspective, these findings highlight the importance of strengthening antenatal surveillance systems and implementing evidence‐based models for maternal risk stratification. Based on WHO recommendations and the local Ministry of Health guidelines, integrated protocols for obstetric–infectious disease management must be reinforced. Establishing regional maternal COVID‐19 registries in Saudi Arabia would also enhance risk prediction and support timely evidence‐based practice.

The present study has some limitations, including its retrospective design and reliance on existing medical records, which may introduce information bias. The lack of a non–COVID‐19 control group also limits causal inference and generalizability.

## 4. Conclusions

This study found that COVID‐19–positive pregnant women experienced adverse outcomes, including preeclampsia, gestational diabetes, preterm birth, and low birth weight. Severe COVID‐19 during pregnancy was strongly associated with higher risks of preeclampsia and ICU admissions for both the mothers and newborns. Maternal comorbidities such as asthma, diabetes, hypertension, and obesity were also linked to poorer outcomes.

## Conflicts of Interest

The authors declare no conflicts of interest.

## Funding

The authors received no specific funding for this work.

## Supporting Information

Supporting Table 1. Relationship between the maternal age and the incidence of adverse maternal and neonatal outcomes in COVID‐19–positive pregnant women (*N* = 138).

Supporting Table 2. Associations between comorbidities and the incidence of adverse maternal and neonatal outcomes in COVID‐19–positive pregnant women (*N* = 138).

## Supporting information


**Supporting Information** Additional supporting information can be found online in the Supporting Information section.

## Data Availability

All data supporting the findings of this study are included within the article.
