# Supplementary material for: Retrospective Cohort Study of Pregnancy Maternal Outcomes of Women With COVID‐19 in King Salman Armed Forces Hospital, Tabuk, Kingdom of Saudi Arabia
Source: Obstet Gynecol Int. 2025 Dec 22;2025:3287655. doi: 10.1155/ogi/3287655 (PMC12767420; doi:10.1155/ogi/3287655)
Supplement: Supplementary file 1 — Supporting Information Additional supporting information can be found online in the Supporting Information section. [file OGI-2025-3287655-s001.docx]

**Supplementary Table 1.** **Relationship between the maternal age and the incidence of adverse maternal and neonatal outcomes in COVID-19 positive pregnant women (N = 138)**

|  | | **Maternal Age (years)** | | **P-value** |
| --- | --- | --- | --- | --- |
|  |  | **Median** | **IQR** |  |
| **Preeclampsia** | No | 30.0 | 24.5-35.0 | 0.004* |
|  | Yes | 34.0 | 27.0-40.0 |  |
| **Gestational diabetes** | No | 30.0 | 25.0-36.0 | 0.949 |
|  | Yes | 30.5 | 25.0-35.0 |  |
| **Placental abruption** | No | 31.0 | 25.0-36.0 | 0.530 |
|  | Yes | 29.0 | 24.0-35.0 |  |
| **PROM** | No | 30.0 | 25.0-36.0 | 0.937 |
|  | Yes | 31.0 | 25.0-36.0 |  |
| **Maternal ICU admission** | No | 30.0 | 25.0-36.0 | 0.279 |
|  | Yes | 36.5 | 32.0-41.0 |  |
| **Mode of delivery** | CS | 32.0 | 27.0-36.0 | 0.008* |
|  | Vaginal | 29.0 | 24.0-35.0 |  |
| **Preterm labor** | No | 30.0 | 25.0-35.0 | 0.149 |
|  | Yes | 33.0 | 25.0-36.0 |  |
| **Low birth weight** | No | 31.0 | 25.0-36.0 | 0.173 |
|  | Yes | 29.0 | 23.0-35.0 |  |
| **Neonatal ICU admission** | No | 30.0 | 25.0-36.0 | 0.928 |
|  | Yes | 32.0 | 24.0-37.0 |  |

*Significant at p<0.05, PROM: premature rupture of membranes, CS: Cesarean section, ICU: intensive care unit, IQR: interquartile range.

**Supplementary Table 2. Associations between comorbidities and** **the incidence of adverse maternal and neonatal outcomes in COVID-19 positive pregnant women (N = 138)**

|  | **Asthma, n (%)** | | **Diabetes Mellitus, n (%)** | | |
| --- | --- | --- | --- | --- | --- |
|  | **No** | **Yes** | **No** | **Yes** | |
| **Preeclampsia** | 5 (4.5%) | 2 (7.7%) | 5 (4.1%) | | 2 (13.3%) |
| **Gestational diabetes** | 4 (3.6%) | 2 (7.7%) | 6 (4.9%) | | 0 (0.0%) |
| **Placental abruption** | 2 (1.8%) | 2 (7.7%) | 3 (2.4%) | | 1 (6.7%) |
| **PROM** | 3 (2.7%) | 1 (3.8%) | 4 (3.3%) | | 0 (0.0%) |
| **Maternal ICU admission** | 1 (0.9%) | 1 (3.8%) | 0 (0.0%) | | 2 (13.3%*) |
| **Mode of delivery, CS** | 57 (50.9%) | 16 (61.5%) | 61 (49.6%) | | 12 (80.0%) |
| **Preterm labor** | 14 (12.5%) | 3 (11.5%) | 14 (11.4%) | | 3 (20.0%) |
| **Low birth weight** | 16 (14.3%) | 5 (19.2%) | 19 (15.4%) | | 2 (13.3%) |
| **Neonatal ICU admission** | 8 (7.1%) | 6 (23.1%*) | 11 (8.9%) | | 3 (20.0%) |
|  | **Hypertension, n (%)** | | **Obesity, n (%)** | | |
|  | **No** | **Yes** | **No** | **Yes** | |
| **Preeclampsia** | 2 (1.6%) | 5 (35.7%*) | 7 (6.5%) | | 0 (0.0%) |
| **Gestational diabetes** | 6 (4.8%) | 0 (0.0%) | 3 (2.8%) | | 3 (9.7%) |
| **Placental abruption** | 3 (2.4%) | 1 (7.1%) | 1 (0.9%) | | 3 (9.7%*) |
| **PROM** | 4 (3.2%) | 0 (0.0%) | 2 (1.9%) | | 2 (6.5%) |
| **Maternal ICU admission** | 0 (0.0%) | 2 (14.3%*) | 1 (0.9%) | | 1 (3.2%) |
| **Mode of delivery, CS** | 63 (50.8%) | 10 (71.4%) | 56 (52.3%) | | 17 (54.8%) |
| **Preterm labor** | 14 (11.3%) | 3 (21.4%) | 12 (11.2%) | | 5 (16.1%) |
| **Low birth weight** | 17 (13.7%) | 4 (28.6%) | 17 (15.9%) | | 4 (12.9%) |
| **Neonatal ICU admission** | 9 (7.3%) | 5 (35.7%*) | 10 (9.3%) | | 4 (12.9%) |

PROM: premature rupture of membranes, CS: Cesarean section, ICU: intensive care unit, * indicates significant association at p < 0.05.
